# Response to the letter to the editor “Methylene blue staining: A novel application to identify the damaged tissues on the surface of pressure ulcers”

**DOI:** 10.1111/iwj.14041

**Published:** 2022-11-30

**Authors:** Chao Lian, Xuan‐Fen Zhang, Xue‐Lei Li, Xiao‐Jun Liu

**Affiliations:** ^1^ Department of Plastic Surgery Lanzhou University Second Hospital Lanzhou China; ^2^ Department of Plastic and Aesthetic Surgery Affiliated Changzhi People's Hospital of Changzhi Medical College Changzhi China; ^3^ Department of Plastic and Aesthetic Surgery Nanfang Hospital of Southern Medical University Guangzhou China

Dear Editor‐in‐Chief,

We greatly appreciate the comments of readers on our recent article “Methylene blue staining: A novel application to identify the damaged tissues on the surface of pressure ulcers.”

Despite great improvements in techniques, precise surgical debridement is still a great challenge for surgeons. Excessive excision may cause loss of viable tissue which frequently leads to an adverse effect whether functional or cosmetic, whereas incomplete debridement leads to necrosis, leaving further residue and ultimately delaying healing.^1^ Even utilising ultrasonic debridement, the damaged tissues can still be inadequately debrided, just because these tissues cannot be visually identified on the wound surface. Methylene blue has been wildly used in medical applications.^2^ Methylene blue is an absorbent dye that oxidises blue and is reduced to colourless. Due to the role of metabolism in living cells, the cell has a strong reducing ability, which can make methylene blue change from blue oxidation to colourless reduction. Therefore, the shade of MBS can also be used as a special dye to identify cell activity. Therefore, we can easily distinguish between non‐viable tissues and viable tissues by the colour change produced by methylene blue staining (MBS). Due to the visualisation of dead or damaged tissues coloured by methylene blue dye, precise surgical debridement of wound could be effectively performed.^3^

According to the readers' perspective, MBS is a simple and effective method to identify the damaged tissues prior to surgical debridement, but there are still four limitations as follows:

First, the sample size in our study was relatively small, which may affect reliability, but this article was published in 2019, and in recent years, we have successfully treated more patients, using MBS as an aid in identifying damaged tissue before surgical debridement of pressure ulcers. In the practice that followed, we expressed great recognition of this method.

The second limitation is the lack of a classification statement on the range of applications of MBS for different types of wounds. In 1980, Davis et al. created the first successful paradigm for burn wound surfaces using MBS as an aid to identify the dead and damaged tissues of before surgery^4^ In 2010, Amir et al. describe their own experience with a technique using MBS to facilitate precise surgical debridement. They used this technique in more than 200 wounds including acute surgical or traumatic wounds, acute and subacute burn wounds, chronic granulating wounds, sinus tracts, and fistulae.^5^ It is probably indicated that MBS‐guided debridement may be applied to most types of wounds. However, to confirm this hypothesis, further investigation is urgently needed.

The third limitation is the lack of classification declaration regarding the application method of MBS for different shapes of wound. Actually, techniques for staining the wound depending on the shape of the wound being debrided. Flat wounds without tunnelling or sinus tracts can be painted and covered using cotton‐tip applicators dipped in methylene blue dye.^3^ But for the surface of wounds that tunnel or contain sinus tracts, such as void‐type pressure ulcer, methylene blue dye was gently forced into the cavity to stain and cover the wound surface using a 5‐mL syringe (Figure 1). it is worth noting that methylene blue dye is not injected into the wound bed or periphery for the purposes of debridement as it is with sentinel lymph node biopsies.^5^ According to the study of Davis et al in 1980, methylene blue (500 mg silver sulphadiazine 1% and 160 mg methylene blue 1%) was applied topically on the dermal surface at least 24 hours before surgery.^3^ Actually, our previous studies were based on this method and most of the patients had achieved an acceptable restoration without significant complications. Despite good clinical efficacy, the optimal concentration of methylene blue still needs further study.

**FIGURE 1:**
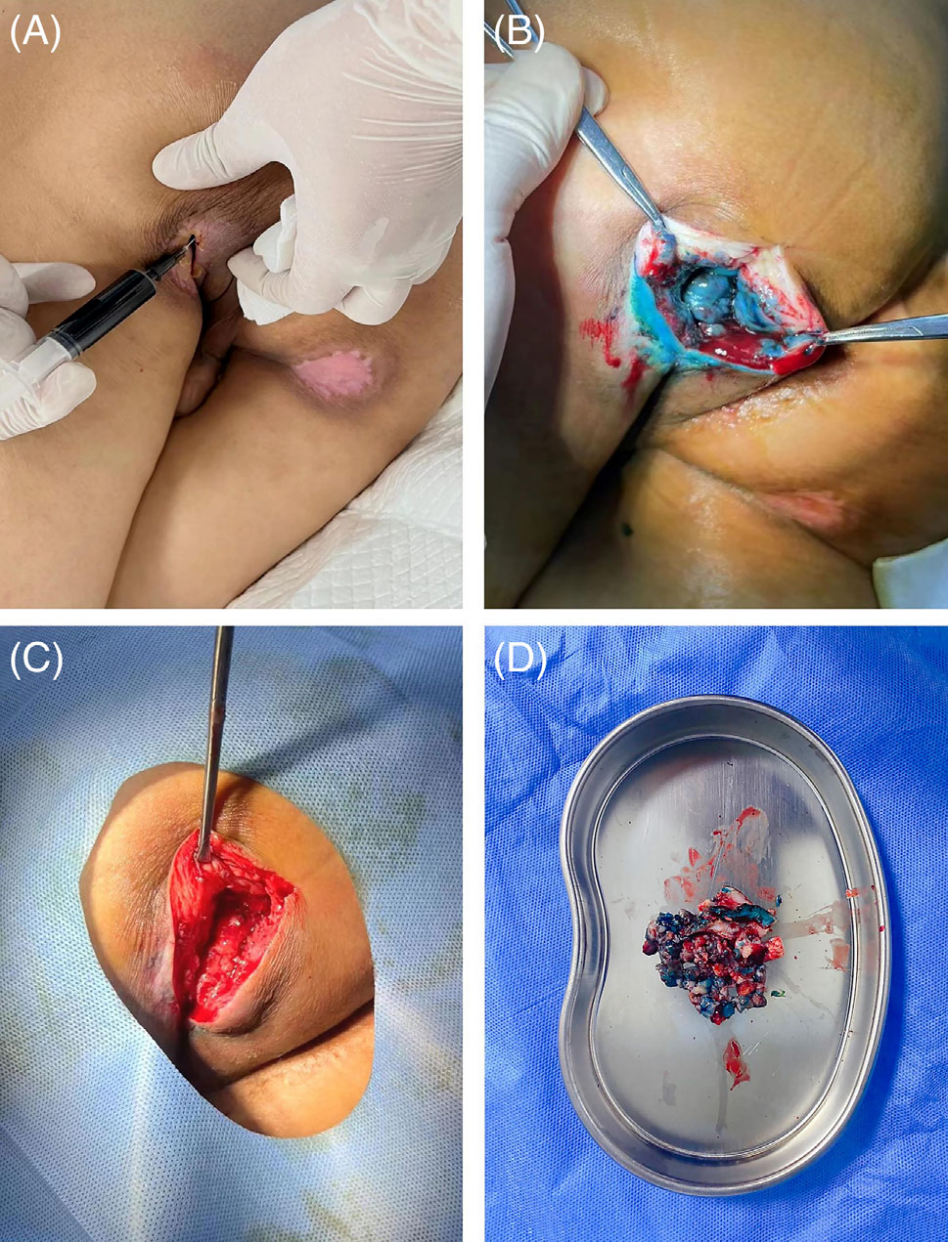
Using methylene blue dye to colour the dead or damaged tissues on the surface of a void‐type pressure sore just prior to surgical debridement. A, Methylene blue dye was gently forced into the cavity to cover the wound surface using a 5‐mL syringe. B, A visible colour change had been produced after 24 h. C, The blue‐staining tissues were totally removed. D, The damaged tissues coloured by methylene blue dye

The last limitation is the lack of elaboration regarding wound closure methods for different wound types. Based on our previous study, herein, we just lightly share our own experience. In general, wound closure methods depend on the general nutritional status and the postoperative defect size. If the patient is well‐nourished and the defect size is relatively small, interrupted vertical mattress suture is the best choice^6^; if the patient is well‐nourished and the defect size is relatively large, the primary choice is one‐stage reconstruction using local advanced or rotation flap^7^; But if the patient is worse‐nourished and the defect size is relatively large, it is strongly recommended that the patient should be treated by a conservative combination therapy instead of reconstructive surgery or direct suturing. Combination application of MBS, ultrasonic debridement, and negative pressure wound therapy is a preferred solution.^8^

For the first time, MBS was successfully applied to the identification of pressure ulcer injury tissue. In summary, using MBS to guide debridement is a simple and effective technique that can be easily applied to most of the chronic wounds and quickly grasped by any surgeons. However, MBS‐guided debridement is not meant to instead of clinical judgement. It is just conducive to guide the doctor's decision on further debridement. To confirm the superiority of MBS‐guided debridement, further investigations are still required.

## Data Availability

Data sharing not applicable to this article as no datasets were generated or analysed during the current study.
